# Supplementary material for: Role and mechanism of NCAPD3 in promoting malignant behaviors in gastric cancer
Source: Front Pharmacol. 2024 Apr 22;15:1341039. doi: 10.3389/fphar.2024.1341039 (PMC11070777; doi:10.3389/fphar.2024.1341039)
Supplement: Supplementary file 11 [file DataSheet2.ZIP › GSEA/immunologic/my_analysis.Gsea.1599462003817/GSE2770_UNTREATED_VS_ACT_CD4_TCELL_48H_UP.html]

Details for gene set GSE2770\_UNTREATED\_VS\_ACT\_CD4\_TCELL\_48H\_UP[GSEA]

|  || Dataset | filtered\_dataset.sample\_info.cls#WT\_versus\_NCAPD3\_MUT |
| Phenotype | sample\_info.cls#WT\_versus\_NCAPD3\_MUT |
| Upregulated in class | WT |
| GeneSet | GSE2770\_UNTREATED\_VS\_ACT\_CD4\_TCELL\_48H\_UP |
| Enrichment Score (ES) | 0.4171442 |
| Normalized Enrichment Score (NES) | 1.9396347 |
| Nominal p-value | 0.0034542314 |
| FDR q-value | 0.7378304 |
| FWER p-Value | 1.0 |
Table: GSEA Results Summary

  

Fig 1: Enrichment plot: GSE2770\_UNTREATED\_VS\_ACT\_CD4\_TCELL\_48H\_UP      
 Profile of the Running ES Score & Positions of GeneSet Members on the Rank Ordered List

  

| SYMBOL | TITLE | RANK IN GENE LIST | RANK METRIC SCORE | RUNNING ES | CORE ENRICHMENT || 1 | 55794 | DDX28 | 68 | 0.856 | 0.0114 | Yes |
| 2 | 11052 | CPSF6 | 89 | 0.814 | 0.0544 | Yes |
| 3 | 92935 | MARS2 | 143 | 0.741 | 0.0684 | Yes |
| 4 | 23450 | SF3B3 | 178 | 0.697 | 0.0931 | Yes |
| 5 | 54919 | DNAAF5 | 184 | 0.690 | 0.1382 | Yes |
| 6 | 145389 | SLC38A6 | 202 | 0.673 | 0.1734 | Yes |
| 7 | 22948 | CCT5 | 215 | 0.663 | 0.2116 | Yes |
| 8 | 127253 | TYW3 | 225 | 0.654 | 0.2512 | Yes |
| 9 | 55599 | RNPC3 | 227 | 0.652 | 0.2966 | Yes |
| 10 | 64794 | DDX31 | 248 | 0.633 | 0.3268 | Yes |
| 11 | 87178 | PNPT1 | 263 | 0.622 | 0.3606 | Yes |
| 12 | 55869 | HDAC8 | 352 | 0.566 | 0.3370 | Yes |
| 13 | 79896 | THNSL1 | 407 | 0.534 | 0.3357 | Yes |
| 14 | 9690 | UBE3C | 470 | 0.493 | 0.3258 | Yes |
| 15 | 10940 | POP1 | 497 | 0.474 | 0.3404 | Yes |
| 16 | 3609 | ILF3 | 544 | 0.449 | 0.3389 | Yes |
| 17 | 27229 | TUBGCP4 | 556 | 0.442 | 0.3622 | Yes |
| 18 | 4548 | MTR | 592 | 0.428 | 0.3672 | Yes |
| 19 | 55181 | SMG8 | 602 | 0.422 | 0.3905 | Yes |
| 20 | 219743 | TYSND1 | 607 | 0.419 | 0.4171 | Yes |
| 21 | 5987 | TRIM27 | 695 | 0.380 | 0.3812 | No |
| 22 | 23076 | RRP1B | 709 | 0.370 | 0.3979 | No |
| 23 | 1736 | DKC1 | 791 | 0.330 | 0.3628 | No |
| 24 | 80230 | RUFY1 | 807 | 0.317 | 0.3743 | No |
| 25 | 11157 | LSM6 | 830 | 0.296 | 0.3794 | No |
| 26 | 6698 | SPRR1A | 1178 | -0.548 | 0.1675 | No |
Table: GSEA details [plain text format]

  

Fig 2: GSE2770\_UNTREATED\_VS\_ACT\_CD4\_TCELL\_48H\_UP      
 Blue-Pink O' Gram in the Space of the Analyzed GeneSet

  

Fig 3: GSE2770\_UNTREATED\_VS\_ACT\_CD4\_TCELL\_48H\_UP: Random ES distribution      
 Gene set null distribution of ES for **GSE2770\_UNTREATED\_VS\_ACT\_CD4\_TCELL\_48H\_UP**
